# Maintenance of prior behaviour can enhance cultural selection

**DOI:** 10.1038/s41598-021-99340-7

**Published:** 2021-10-06

**Authors:** Bradley Walker, José Segovia Martín, Monica Tamariz, Nicolas Fay

**Affiliations:** 1 School of Psychological Science, University of Western Australia, 35 Stirling Highway, Crawley, WA 6009 Australia; 2 Centre National de la Recherche Scientifique (CNRS), Institut des Systèmes Complexes Paris Île-de-France (ISC-PIF), Paris, France; 3 Department of Psychology, Heriot-Watt University, Edinburgh, UK

**Keywords:** Cultural evolution, Human behaviour

## Abstract

Many cultural phenomena evolve through a Darwinian process whereby adaptive variants are selected and spread at the expense of competing variants. While cultural evolutionary theory emphasises the importance of social learning to this process, experimental studies indicate that people’s dominant response is to maintain their prior behaviour. In addition, while payoff-biased learning is crucial to Darwinian cultural evolution, learner behaviour is not always guided by variant payoffs. Here, we use agent-based modelling to investigate the role of maintenance in Darwinian cultural evolution. We vary the degree to which learner behaviour is payoff-biased (i.e., based on critical evaluation of variant payoffs), and compare three uncritical (non-payoff-biased) strategies that are used alongside payoff-biased learning: copying others, innovating new variants, and maintaining prior variants. In line with previous research, we show that some level of payoff-biased learning is crucial for populations to converge on adaptive cultural variants. Importantly, when combined with payoff-biased learning, uncritical maintenance leads to stronger population-level adaptation than uncritical copying or innovation, highlighting the importance of maintenance to cultural selection. This advantage of maintenance as a default learning strategy may help explain why it is a common human behaviour.

## Introduction

Many human traits, including language, religion and cooking, are culturally inherited^1–3^. We acquire these traits through social learning, and they evolve over time^4–6^. In many cases culture evolves through a Darwinian process where more adaptive variants selectively spread through the population at the expense of less adaptive variants^7^. Historical examples include the evolution of stone tools^8^, urban legends^9^, violin sound holes^10^, and aeroplane landing gear^11^. Experimental simulations have been used to demonstrate Darwinian cultural evolution under controlled laboratory conditions. Here, small groups of human participants produce material or symbolic artefacts such as stone tools^12^, arrowheads^13–15^, paper planes^16,17^ and communication systems^18–20^ that adaptively improve over time.

When learning in these studies, and in general, people can switch between three basic strategies: they can copy a variant from someone else (copying), innovate a new variant (innovation), or reuse a prior variant (maintenance). Whereas cultural evolutionary theory emphasises the importance of social learning (i.e., copying)^1,4,5,21^, a striking finding from experimental studies is that people’s dominant response is to maintain their prior behaviour^22–25^ (also known as egocentric discounting^22,26,27^ and behavioural conservatism^28–30^). Researchers have struggled to explain this^27^. Given the dominance of maintenance in human behaviour, it is important to examine the role it might play in Darwinian cultural evolution. If maintenance confers a benefit over other learning strategies, this may help to explain why it is common.

One reason people might maintain a prior variant is because it has a higher payoff than other variants. This can be considered a form of payoff-biased learning, a term typically applied in the context of social learning^31–33^ (i.e., copying based on variant payoffs). Asocial learning can also be considered payoff-biased, in the sense that it involves switching between innovation and maintenance based on variant payoffs (i.e., the “error” in “trial and error”). Mathematical and computational modelling have shown that payoff-biased learning is necessary for the selective spread of adaptive variants in a population^34–38^. These models show that payoff-biased learning increases the adaptiveness of culture through *adaptive filtering*^35^: learners critically evaluate the variants they encounter, and are guided by a payoff bias to adopt adaptive variants and discard maladaptive variants. This filters maladaptive variants out of the population, increasing the probability that the variants that spread through social learning will be adaptive.

While payoff-biased learning is necessary for adaptive culture, variant adoption is not always guided by payoffs: a significant proportion of people maintain their own variants despite evaluating other variants as having higher payoffs^26,39^. Furthermore, there is evidence that people do not always evaluate variant payoffs, declining to even access the variants produced by others^40^. One reason for this might be that payoff evaluation is costly: there may be health risks involved (e.g., assessing an unfamiliar power tool), and learners do not always have the resources available, in terms of time, energy, motivation or expertise. This is recognised in cultural evolutionary theory^4,41,42^, which includes low-cost strategies for indirectly estimating payoffs based on features such as model prestige^43,44^ or majority size^45,46^. However, these low-cost strategies are not used reliably^32,47,48^, and researchers have overlooked uncritical strategies that incur no evaluation cost at all. Uncritical strategies alone cannot lead to adaptive culture, but if learning is sometimes payoff-biased overall costs may be reduced by reverting to uncritical “default” strategies (i.e., arbitrarily copying, innovating or maintaining without estimating payoffs; see Tamariz^49^). There is some evidence that uncritical maintenance plays a role in cultural selection during the development of novel communication systems^50^, though it remains unclear how Darwinian cultural evolution would be impacted by different uncritical strategies.

In the present paper we use agent-based modelling to test how Darwinian cultural evolution may be impacted by maintenance, in comparison to copying and innovation, when varying the degree to which learners are critical (i.e., the degree to which they engage in payoff-biased learning). To test the robustness of our results, we examine unstructured populations with a range of sizes (10, 20, 100 and 200 agents), as well as structured 200-agent populations with a range of neighbourhood sizes (neighbourhoods of approximately 6, 16, 25 and 33 agents). In line with previous research, we show that some level of payoff-biased learning is crucial for populations to converge on adaptive variants. Importantly, when learning is not always critical, a default strategy to uncritically maintain prior behaviour improves the cultural selection process relative to uncritical copying and innovation.

## Method

### Purpose

The purpose of the model is to understand how Darwinian cultural evolution is affected by the degree to which learners are critical, adopting variants in a payoff-biased manner, and by whether they copy, innovate or maintain when uncritical. We compare variant diversity and adaptation across homogeneous populations of agents who copied when uncritical, innovated when uncritical or maintained when uncritical.

### The model

The model consists of populations of agents, characterised by the variables shown in Table 1. On each of a series of 200 time steps, the task for each agent in a population was to choose a cultural variant to carry into the next time step (agents possessed one variant at a time, with no memory of earlier variants). There were three sources of variants: agents could (1) copy the variant possessed by another agent in their population (i.e., a model), (2) innovate a new variant, or (3) maintain their variant from the previous time step. Agents chose between copying, innovation and maintenance based on variant payoffs (“Variants and variant payoffs” section) when they were critical (i.e., guided by payoff bias, “Payoff bias” section). When uncritical, the choice was determined by an agent’s learner type (“Learner type” section). There was also a possibility of producing the wrong variant through error (error rate, “Error rate” section). To test the robustness of the results, we simulated populations in a variety of sizes and with different neighbourhood structures (“Population size and structure” section).

**Table 1:** Model parameters and variables.

Parameter/variable	Symbol	Value(s)
Payoff bias	*b*	0, 0.2, 0.4, 0.6, 0.8, 1
Learner type	*t*	Copier, innovator, maintainer
Error rate	*e*	0.02
Variant pool size (number of possible variants)		100
Population size		10, 20, 100, 200
Population structure		Random, neighbourhoods of approximately 6, 16, 25, 33 agents
Time steps		200

#### Process overview

At the start of each run, a population was generated with a variant pool (100 variants with associated payoffs, representing all possible variants) and a list of agents. All the agents in the population were initialised with a random variant sampled with replacement from the population’s variant pool. All agents in the population were given the same payoff bias, learner type and error rate parameter values.

**Figure 1:**
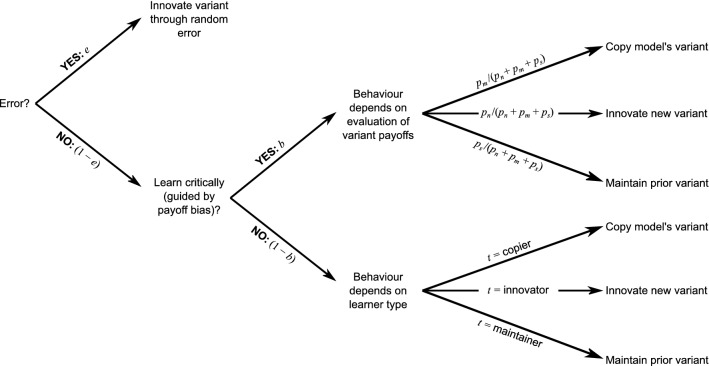
Diagram showing how the various parameters drove agents’ variant choices.

At each of 200 time steps, each agent in the population was given the opportunity to innovate a new variant, copy the variant of another agent in the population (a model), or maintain their prior variant (or make an error and adopt a random variant). This process was sequential (i.e., a later agent could copy from an earlier agent whose variant had already changed during that time step), and the same model could be selected by multiple agents. The structure of agents’ choices, as determined by the model parameters, is shown in Fig. 1. Payoff bias *b*, learner type *t*, and error rate *e* were set when running the simulations, while the payoff of the new, innovated variant *p*_*n*_, the payoff of the model’s variant *p*_*m*_, and the payoff of the agent’s own variant *p*_*s*_ depended on the population’s payoff distribution. The equations that determined agent behaviour from these parameters are shown in Supplementary Information S1.

#### Variants and variant payoffs

Each population was initialised with a variant pool containing 100 variants, representing all possible variants. Agents’ variants were sampled from this pool. Following Rendell et al.^51^, variant payoffs were determined by an exponential distribution. This type of distribution is considered realistic, as it gives a small number of variants with very high payoffs, and a majority of variants with (very) low payoffs. Each variant in the pool was represented arbitrarily by an integer between 0 and 99, and was associated with a payoff determined by sampling a random value from an exponential distribution (λ = 1; as per Rendell et al., values were then squared, doubled and rounded, and 1 was added to avoid division by zero errors; this gave a minimum payoff of 1 and a maximum payoff typically around 50). New variant payoffs were generated for each run.

#### Payoff bias

The payoff bias parameter (*b*) varied over 6 levels, in increments of 0.2 from 0 (fully uncritical) to 1 (fully critical). This parameter represented the probability with which an agent would behave critically, choosing between variants based on their evaluated payoffs (e.g., 20% of the time when payoff bias was 0.2). The model was agnostic with respect to whether agents evaluated payoffs directly (e.g., by experimentation) or indirectly (e.g., by estimating others’ payoffs or through forward modelling). When agents were guided by payoff bias, the probability of choosing each variant was proportional to the variant’s payoff. For instance, given variants with payoffs of 10, 6 and 4, the variant with payoff 10 would be chosen with a probability of 0.5 (i.e., 10/(10 + 6 + 4))^52^. When agents were not guided by payoff bias (i.e., were uncritical), their behaviour was determined by their learner type.

#### Learner type

The learner type parameter (*t*) defined agents’ uncritical default behaviour, so that a learner could either be a copier, an innovator, or a maintainer (though note that learner type did not vary within populations). When payoff bias was 0, agent behaviour was never guided by variant payoffs, and was fully dictated by learner type: a copier would always copy, an innovator would always innovate, and a maintainer would always maintain their prior variant, regardless of variant payoffs (this simulated cultural drift under the different learner types). When payoff bias was 1, agent behaviour was always based on variant payoffs and learner type never came into play, so copiers, innovators and maintainers behaved identically under this parameter setting. At intermediate payoff bias levels, agents would be guided by payoff bias some of the time and at other times revert to the default behaviour determined by their learner type.

#### Error rate

The error rate parameter (*e*) represented all kinds of errors that could lead a learner to produce the wrong variant (e.g., copying error, production error), and was set at 0.02, as this was the error rate in past cultural transmission experiments and used in previous models^50,53,54^. When agents produced the wrong variant through error, they adopted a variant randomly sampled from the population’s variant pool (including the variant they were trying to produce).

#### Population size and structure

**Figure 2:**
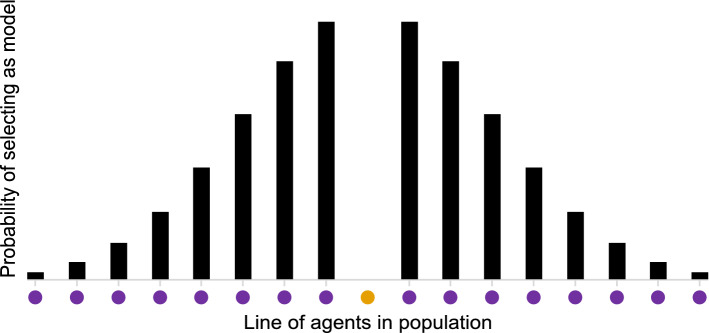
Diagram showing how neighbourhood structure was imposed by selecting each agent’s (in orange) models from the neighbouring agents (in purple) using Gaussian distributions.

We first simulated populations of various sizes, with 10, 20, 100 and 200 agents each. These populations were unstructured, with agents’ models (for the purposes of copying) selected at random from the whole population. As large unstructured populations are unrealistic, and to test the robustness of our results, we next imposed structure on populations of 200 agents by selecting models from the “neighbourhood” around each agent using Gaussian distributions, following Barr^55^. The agents were treated as being evenly distributed on a line, and a model was selected by sampling from a Gaussian distribution centred on an agent (and not allowing an agent to be their own model), so that increased distance from an agent reduced the probability of being selected as their model (see Fig. 2). The line was assumed to be circular (so the first and last agents in the population were neighbours). We used Gaussian distributions with standard deviations of 1, 3, 5 and 7 to create neighbourhood sizes of approximately 6, 16, 25 and 33 agents respectively, representing the number of models an agent had access to over the simulated time period.

### Quantifying variant diversity and adaptation

To measure the effect of payoff-biased learning and the default uncritical behaviours on Darwinian cultural evolution, population-level variant diversity and average payoff were measured at the end of each time step. For variant diversity, we used the Gini-Simpson index of diversity^56^, an ecological measure previously applied to culture^57–59^. With this measure, scores close to 1 indicate high diversity (agents using different variants) and a score of 0 indicates no diversity (all agents sharing the same variant). To calculate a population’s average payoff, we simply averaged the payoffs of variants possessed by the agents in the population. When the average payoff increased over time, we considered a population to be demonstrating adaptation.

## Results

The first set of simulations examined the influence of the different parameter combinations on cultural variant diversity and adaptation in populations of different sizes (10, 20, 100 and 200 agents). The populations were unstructured: every agent in the population had an equal probability of being selected as a model for the purposes of copying. We ran 10,000 simulations for each parameter combination.

**Figure 3:**
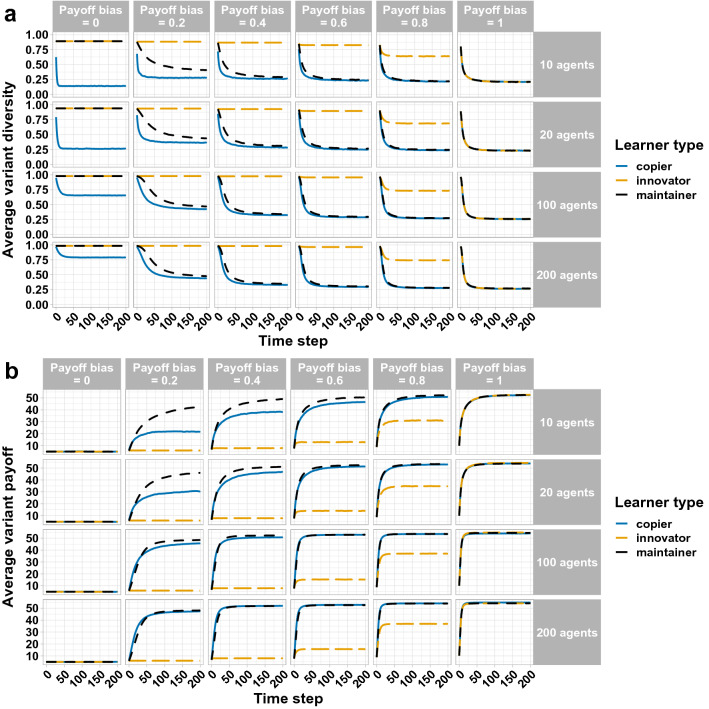
Change﻿ in cultural diversity (**a**) and average variant payoff (adaptation) (**b**) over 200 time steps as a function of the different parameter combinations (payoff bias, learner type and population size). Populations were unstructured. Variant diversity was measured by the Gini-Simpson index of diversity (maximum for 10-, 20-, 100- and 200-agent populations = 0.9, 0.95, 0.99 and 0.995 respectively). Each line is the average of 10,000 simulations.

Variant diversity decreased over time as agents converged on a reduced set of cultural variants. Convergence was faster and stronger as payoff bias increased. Smaller populations showed the strongest convergence (though not necessarily on the most adaptive variants). Population-level convergence was moderated by learner type: copiers converged most strongly, with maintainers retaining more diversity, and innovators continually generating more diversity and converging the least (see Fig. 3a).

Variant adaptation required payoff-biased learning. As payoff bias increased, populations increasingly converged on the best-adapted (highest payoff) cultural variants. Increased population size increased the rate of convergence on the best-adapted variants, as larger populations had higher initial variation to select from. Crucially, population-level adaptation was moderated by learner type: it was enhanced in populations of maintainers, and impeded in populations of copiers and (more strongly) innovators. The benefit of a maintenance strategy on population-level adaptation was most strongly seen with low-to-moderate payoff bias in smaller populations (see Fig. 3b).

The first set of simulations showed a stronger effect of learner type in small unstructured populations than in large unstructured populations. However, large unstructured populations are not realistic: people socialise in smaller subgroups within larger populations. To address this concern, and to test the robustness of our results, the second set of simulations examined the influence of the different parameter combinations on variant diversity and adaptation in structured populations with neighbourhood sizes of approximately 6, 16, 25 and 33 agents. Population size was held constant at 200 agents. We ran 10,000 simulations for each parameter combination.

**Figure 4:**
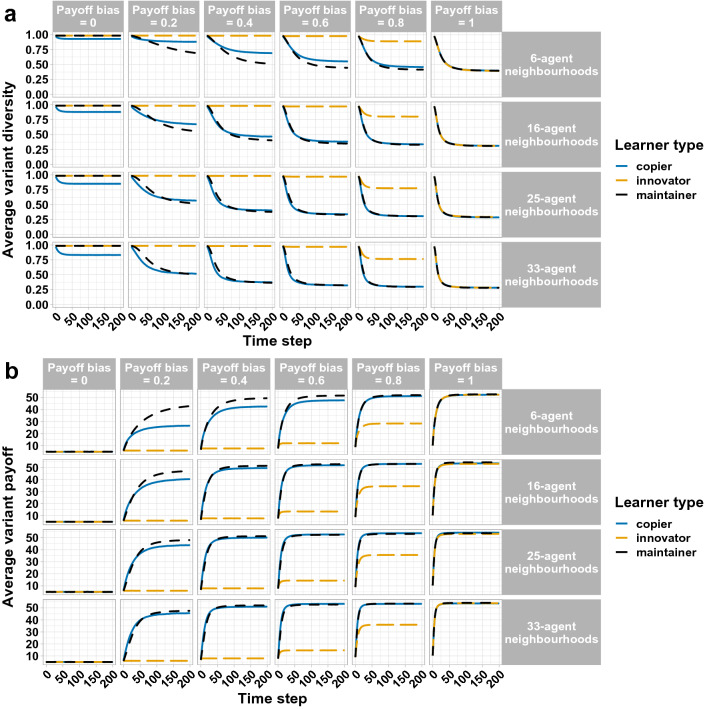
Change in cultural diversity (**a**) and average variant payoff (adaptation) (**b**) over 200 time steps as a function of the different parameter combinations (payoff bias, learner type and neighbourhood size). The number of agents in each neighbourhood is approximate (see “Method” section). Each population had 200 agents. Variant diversity was measured by the Gini-Simpson index of diversity (maximum for 200-agent populations = 0.995). Each line is the average of 10,000 simulations.

Results from the population structure simulations largely mirror the results from the population size simulations. Agents converged on a reduced set of cultural variants over time, and convergence was faster and stronger as payoff bias increased. Populations with larger neighbourhoods showed the strongest convergence (consistent with Barr^55^). Population-level convergence was again moderated by learner type. Again, populations of innovators converged the least; however, in contrast to the unstructured populations, in the structured populations maintainers converged to a similar or higher level than copiers (see Fig. 4a).

Variant adaptation required payoff-biased learning. As payoff bias increased, populations increasingly converged on the best-adapted cultural variants. Increased neighbourhood size increased the rate of convergence on the best-adapted variants, as larger neighbourhoods granted access to more variation to select from. Crucially, population-level adaptation was moderated by learner type: it was enhanced in populations of maintainers, and impeded in populations of copiers and (more strongly) innovators. The benefit of a maintenance strategy on population-level adaptation was most strongly seen with low-to-moderate payoff bias in populations with smaller neighbourhoods (see Fig. 4b).

## Discussion

During social interaction people encounter the cultural variants produced by others, and can compare these to their own cultural variants. A similar process occurs during asocial learning, where people can compare innovated variants to those they already possess. Selection occurs when people adopt one variant and discard the other. This is the premise of the simulations reported in the present paper, where we examined the population-level outcomes of this selection process. In line with previous research, we demonstrated that population-level adaptation required agents to be critical, relying on some level of payoff-biased learning^34–37^. Population-level adaptation also benefited from greater access to cultural variation via increased population size or neighbourhood size^17,60,61^. Greater access to cultural variation increased the probability of agents encountering better-adapted variants, and payoff-biased learning increased the probability of these variants being copied and thereby propagating within the population (see also Schlag^52,62^). When learners were uncritical and did not base their variant choices on payoffs, copying accelerated convergence by eliminating cultural variation. This involved occasionally overwriting better-adapted variants with worse ones. Uncritical innovation also had this problem, and additionally led to the introduction (or reintroduction) of low-payoff variants. Thus copying and innovation as uncritical default strategies disrupted or even reversed the adaptive filtering process. By contrast, uncritical maintenance paused adaptive filtering without disrupting it; cultural variation was retained, slowing convergence, but increasing the retention of better-adapted cultural variants. This improved the environment from which payoff-biased learning could pick out the best-adapted cultural variants, leading to stronger population-level adaptation.

When payoff bias was strongest variant selection was unaffected by the uncritical default behaviours (copying, innovating and maintaining). Weaker payoff bias allowed the default behaviours to influence variant choice. This is why the biggest benefit of maintenance over copying and innovation (and the biggest benefit of copying over innovation) occurred when payoff bias was weak-to-moderate (which may also be more realistic than a stronger level of payoff bias^50^). The benefit of maintenance over copying and innovation was most evident in small populations and large populations with small neighbourhood sizes. The loss of adaptive variants due to uncritical copying or innovation was especially detrimental in small populations due to the lower probability of other agents having a “replacement” copy of the discarded variant. Variant replacement was less problematic in larger populations because multiple copies were more likely to co-exist. However, in large populations organized into small neighbourhoods, agents had less access to variants and therefore also ran into the variant replacement problem. This is why population-level adaptation was stronger for maintainers than for copiers or innovators. It is worth noting that the effects of population and neighbourhood size are dependent on the number of possible variants; when the number of possible variants is increased the benefit of maintenance extends to the larger population and neighbourhood sizes (see Supplementary Information S2). So, while the population and neighbourhood sizes we simulated can be considered “large” or “small” relative to the number of possible variants (i.e., 100), the absolute sizes cannot be interpreted straightforwardly.

An unexpected finding was that populations of maintainers converged as much, or more than, populations of copiers when population structure was introduced. By contrast, in unstructured populations copiers converged more than maintainers. This difference in convergence can be explained by a combination of uncritical copying and neighbourhood structure restricting the spread of high-payoff variants. In structured populations, uncritical copying could create “pockets” of low-payoff variants. For agents within these pockets, the variants they had access to via maintenance and copying often had lower payoffs than the variants they had access to through innovation. This increased the innovation rate when agents were critical, reintroducing variation into the population and impeding convergence. Unstructured populations of copiers did not experience this to the same degree, as the spread of high-payoff variants from elsewhere in the population was unrestricted and low-payoff pockets were less likely to form.

Although maintenance of prior behaviour is common in studies of social learning, researchers have struggled to explain why this is the case^27^. For instance, some researchers have suggested that people may prefer their own position because they have more complete access to the reasoning behind it^26,63^; however, people overweight positions falsely presented as their own, for which they have no access to the reasoning^64^. Other explanations, such as anchoring effects and culture-specific individualistic values, have been similarly unsupported^27^. Our results suggest another line of explanation: maintenance could be adaptive. More specifically, our simulations suggest that maintenance is safer than copying or innovation when not basing variant choices on critical evaluation. This could support an evolutionary explanation for the ubiquity of maintenance (e.g., where natural selection favours defaulting to maintenance when evaluation is costly), but could also indicate other, more proximate explanations; for instance, if maintenance is adaptive then asocial learning may lead people to rely on it. Future research could explore this line of explanation.

A potential limitation of the reported simulations is that we did not consider any costs that may be incurred by learners (e.g., researchers typically consider asocial learning to be costlier than social learning). We address two types of cost in the Supplementary Information. First, we considered how Darwinian cultural evolution may be affected by an evaluation cost (Supplementary Information S3), which is applied whenever a learner adopts a variant through payoff-biased learning. While payoff-biased learning was again needed for cultural adaptation, when evaluation was costly any extra benefit of more-than-minimal payoff-biased learning was overwhelmed by the cost incurred. As a result, the optimal level of payoff bias in the simulations was low—the same level at which maintainers had the largest advantage over copiers and innovators across all simulations. Second, we considered how Darwinian cultural evolution may be affected by a learning cost (Supplementary Information S4), which is applied whenever a learner adopts a variant through copying or innovation (i.e., where the variant is new so there are costs to learning it, such as time and energy, and the learner has reduced proficiency). Stronger learning costs increased the advantage of maintainers over copiers and innovators, and increased the amount of maintenance occurring in all populations via payoff-biased learning. These results demonstrate that incorporating two types of cost into the simulations boosts the advantage of maintenance over copying and innovation as a default learning strategy.

## Conclusion

Maintenance of prior behaviour, as opposed to copying or innovation, is common during social learning, but has been overlooked in the social learning literature^24^ and researchers have struggled to explain its dominance^27^. The agent-based simulations reported here demonstrate that, when learners do not rely exclusively on critical, payoff-biased learning, maintenance as a “default” uncritical strategy can enhance population-level adaptation compared to copying and innovation. Copying and innovation as uncritical default strategies disrupted the adaptive filtering process underlying Darwinian cultural evolution, by overwriting adaptive variants and introducing maladaptive variants. By contrast, maintenance paused adaptive filtering without disrupting it, retaining filtered variation until it could be further filtered by payoff-biased learning. This led to stronger population-level adaptation, especially when payoff bias was weak-to-moderate and agents were in small populations or large populations organised into small neighbourhoods. These results demonstrate the effect that default uncritical strategies may have on Darwinian cultural evolution. In addition, the benefit of maintenance as a default strategy may explain its dominance in human social learning.

## Supplementary Information

Supplementary Information.

## Data Availability

The simulation code and data generated by it are available on the Open Science Framework: https://osf.io/6hmg5/?view_only=7dc34448fb774969b99440ba9724ab56.
