# Supplementary material for: Maintenance of prior behaviour can enhance cultural selection
Source: Sci Rep. 2021 Oct 6;11:19897. doi: 10.1038/s41598-021-99340-7 (PMC8494921; doi:10.1038/s41598-021-99340-7)
Supplement: Supplementary file 1 — Supplementary Information. [file 41598_2021_99340_MOESM1_ESM.pdf]

Maintenance of Prior Behaviour Can Enhance Cultural Selection

Bradley Walker, José Segovia Martín, Monica Tamariz, & Nicolas Fay

Supplementary Information

S1. Equations determining agent behaviour

The parameters governing agent behaviour were payoff bias b, learner type t, and error rate e (set when running the simulations), as well as the payoff of the innovated variant p_n, the payoff of the model's variant p_m, and the payoff of the agent's own variant p_s (determined by the population's specific payoff distribution). For the equations, the parameter t further defined three variables: t_c, the probability of copying when uncritical, set to 1 for copiers and 0 for innovators and maintainers; t_i, the probability of innovating when uncritical, set to 1 for innovators and 0 for copiers and maintainers; and t_m, the probability of maintaining when uncritical, set to 1 for maintainers and 0 for innovators and copiers.

The probability that an agent would adopt the wrong variant through error was determined by the parameter e. The probability that an agent would copy their model's variant, either based on its payoff or because the agent was a copier, was determined by Supplementary Equation S1.

$$(1-e)\left((1-b)(t_c)+b\left(\frac{p_m}{p_n+p_m+p_s}\right)\right) \quad \text{(S1)}$$

The probability that an agent would adopt the innovated variant they had randomly sampled from the variant pool, either based on its payoff or because the agent was an innovator, was determined by Supplementary Equation S2.

$$(1-e)\left((1-b)(t_i)+b\left(\frac{p_n}{p_n+p_m+p_s}\right)\right) \quad \text{(S2)}$$

The probability that an agent would maintain their own variant, either based on its payoff or because the agent was a maintainer, was determined by Supplementary Equation S3.

$$(1-e)\left((1-b)(t_m)+b\left(\frac{p_s}{p_n+p_m+p_s}\right)\right) \quad \text{(S3)}$$

S2. Larger variant pool

Here we simulated 200-agent populations (structured and with 33-agent neighbourhoods) where the variant pool size (i.e., number of possible variants) was set to 10,000. With variant pool size set to 100 in the main paper, the difference between maintainers and copiers was minimal in these populations. With variant pool size increased, the pattern of results here is more comparable to the previously simulated smaller populations, or large populations with smaller neighbourhood sizes: populations of copiers converged more quickly than populations of maintainers (see Supplementary Figure S1a), and population-level adaptation was enhanced in populations of maintainers relative to copiers (see Supplementary Figure S1b). Increasing the variant pool size also increased the maximum payoff in most populations (to around 185), as a natural outcome of the way variant payoffs were sampled from an exponential distribution.

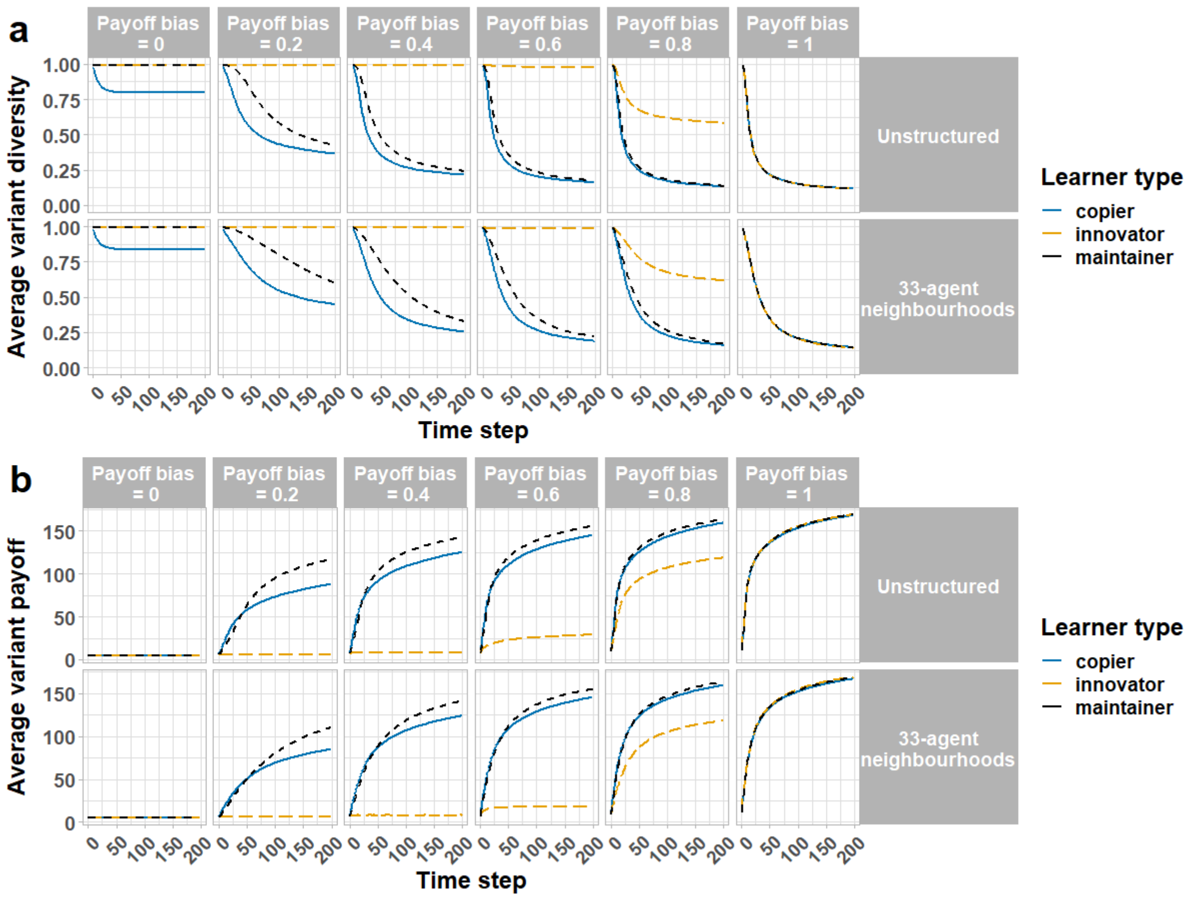

Supplementary Figure S1. Change in cultural diversity (a) and average variant payoff (adaptation) (b) over 200 time steps as a function of the different parameter combinations (payoff bias, learner type and population structure). Each population had 200 agents, and there were 10,000 possible variants. Variant diversity was measured by the Gini-Simpson index of diversity (maximum for 200-agent populations = .995). Each line is the average of 10,000 simulations.

S3. Evaluation cost

Here we simulated unstructured populations of 100 agents, varying an evaluation cost parameter. Evaluation cost was applied whenever an agent engaged in payoff-biased learning (i.e., innovated, copied or maintained based on an evaluation of variant payoffs). When the payoff of an agent's variant was calculated, it was multiplied by 1 minus evaluation cost. So, for example, an evaluation cost of .75 meant that the payoff would be reduced by 75%. We examined evaluation costs of 0 (i.e., no evaluation cost, as in the main paper), .25, .5, .75 and 1. Evaluation cost did not affect agents' payoff evaluations, as the relative payoffs of the variants being considered would be unchanged (e.g., 10 is twice as large as 5 whether or not you multiply them both by .25). As agents could not choose whether or not to use payoff-biased learning in order to maximise variant payoffs (their only choice was between variants when already using payoff-biased learning), they could not strategically avoid evaluation costs and so these did not affect their variant choices; consequently, variant diversity was unaffected by evaluation cost (see Supplementary Figure S2a).

However, evaluation cost did reduce adaptation (see Supplementary Figure S2b). As evaluation cost and payoff bias increased, the cost accumulated and the average payoff of variants in the population decreased. For populations of copiers and maintainers, the optimal level of payoff bias was low when evaluation cost was non-zero: a small amount of payoff-biased learning was needed in order for populations to converge on adaptive variants (in fact, the same amount that gave the largest advantage for maintainers in the main paper), but any advantage of further increases was offset by the evaluation cost. In populations of innovators, adaptation was so low and dependent on payoff-biased learning that higher payoff bias increased payoffs more than the attendant increase in evaluation cost reduced it, and so maximal payoff bias was still optimal in these populations.

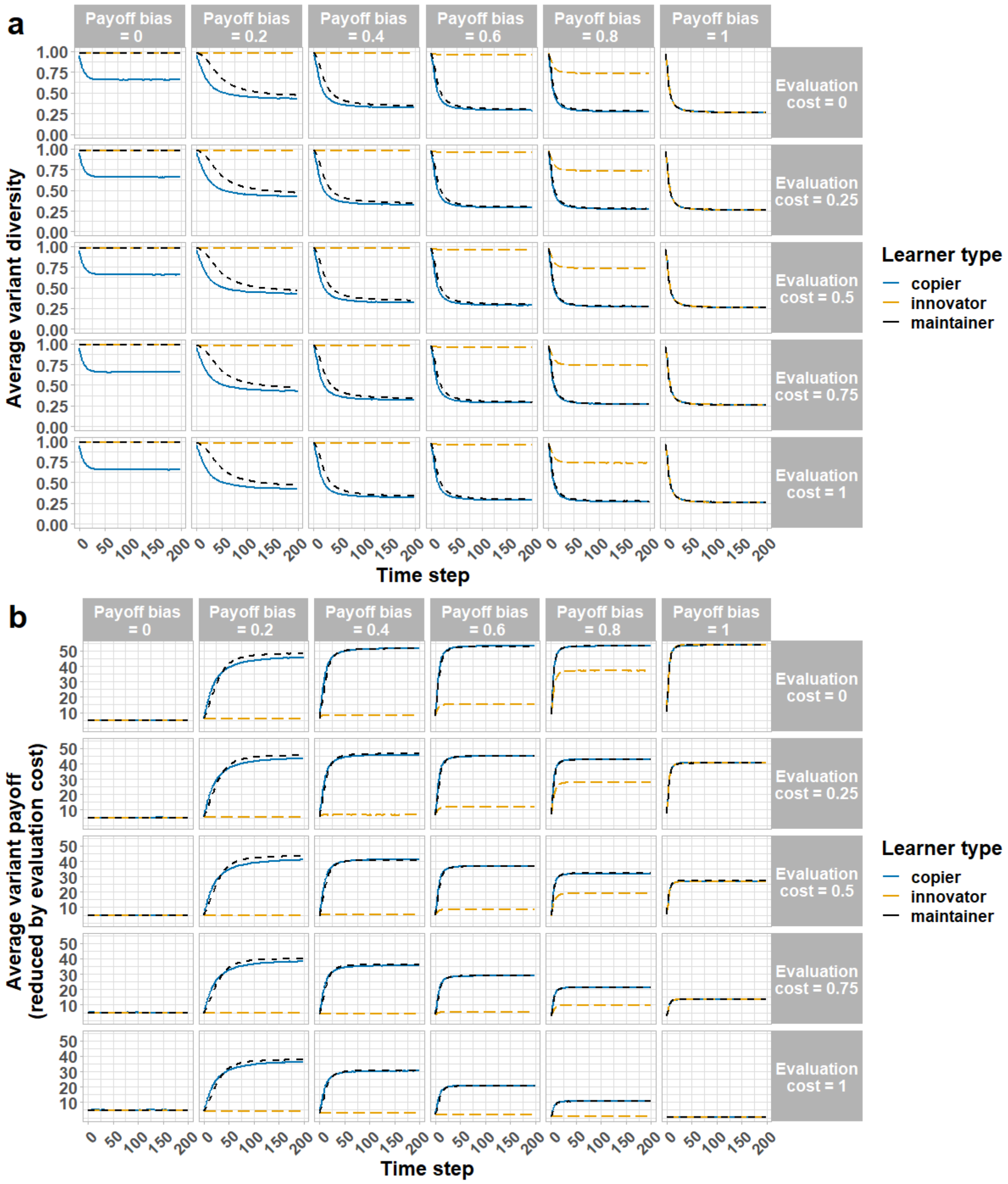

Supplementary Figure S2. Change in cultural diversity (a) and average variant payoff (adaptation) (b) over 200 time steps as a function of the different parameter combinations (payoff bias, learner type and evaluation cost). Each population had 100 agents and was unstructured. Variant diversity was measured by the Gini-Simpson index of diversity (maximum for 100-agent populations = .99). Each line is the average of 10,000 simulations.

S4. Learning cost

Here we simulated unstructured populations of 100 agents, varying a learning cost parameter. Learning cost was applied whenever an agent copied or innovated (i.e., adopted a new variant; this included innovation through error). As with evaluation cost, when the payoff of an agent's variant was calculated, it was multiplied by 1 minus learning cost (so, e.g., a learning cost of .75 meant that the payoff would be reduced by 75%). We examined learning costs of 0 (i.e., no learning cost, as in the main paper), .25, .5, .75 and 1. Learning cost was also included in agents' payoff evaluations, so they could anticipate that their ability to extract the full payoff from a new variant would be reduced.

When agents were critical (payoff bias > 0), convergence was reduced as learning cost increased (most noticeably when the cost was 1; see Supplementary Figure S3a). This is because payoff-biased learning favoured maintenance more as learning cost increased, since only the agent's prior variant escaped the cost. When the learning cost was total only populations of copiers converged at all, and in this case increased payoff bias reduced convergence (in contrast to the increasing effect when learning cost was lower), due to the fact that more variant choices were made on the basis of payoff evaluations, meaning that the agents maintained more often.

As learning cost increased, adaptation was reduced (see Supplementary Figure S3b). When learning cost was total, adaptation became impossible because any alternative to an agent's initial variant had an anticipated payoff of zero. When learning cost was not total, the effect of the cost on maintainers was less than the effect on copiers and innovators, who adopted new variants more often. The benefit of being a maintainer was highest when payoff bias was low, as copiers and innovators could only maintain (and so avoid learning costs) when they engaged in payoff-biased learning. Stronger learning costs, like smaller population and neighbourhood sizes, increased the benefit of a maintenance strategy over copying and innovation.

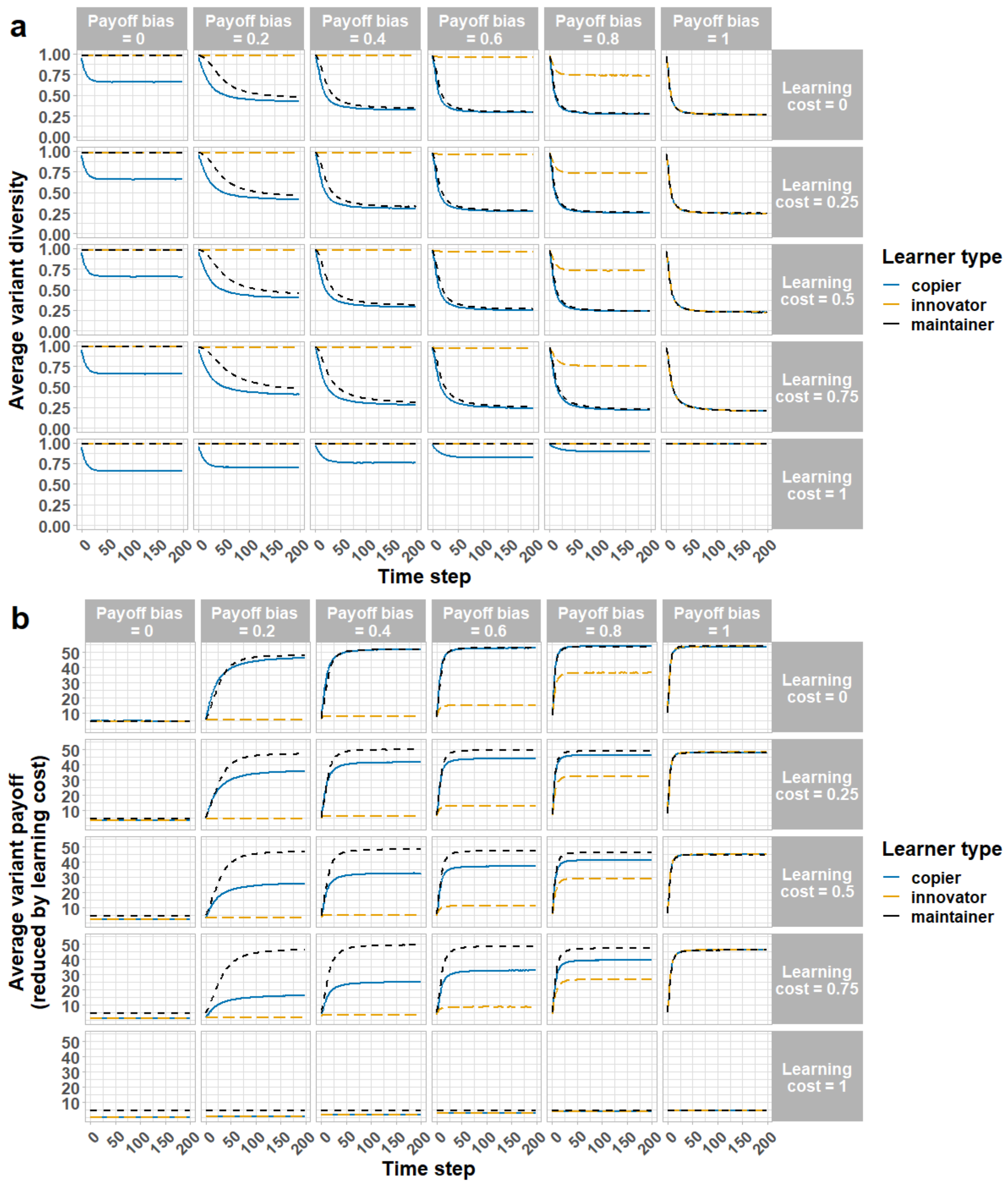

Supplementary Figure S3. Change in cultural diversity (a) and average variant payoff (adaptation) (b) over 200 time steps as a function of the different parameter combinations (payoff bias, learner type and learning cost). Each population had 100 agents and was unstructured. Variant diversity was measured by the Gini-Simpson index of diversity (maximum for 100-agent populations = .99). Each line is the average of 10,000 simulations.
